# Effects of supplementation with narasin, salinomycin, or flavomycin on performance and ruminal fermentation characteristics of *Bos indicus* Nellore cattle fed with forage-based diets

**DOI:** 10.1093/jas/skab005

**Published:** 2021-04-16

**Authors:** Arnaldo Cintra Limede, Rodrigo S Marques, Daniel Montanher Polizel, Bruno Ieda Cappellozza, Alexandre Arantes Miszura, José Paulo Roman Barroso, André Storti Martins, Lairana Aline Sardinha, Marcelo Baggio, Alexandre Vaz Pires

**Affiliations:** 1 Department of Animal Nutrition and Animal Production, University of São Paulo, Pirassununga 13635-000, Brazil; 2 Department of Animal and Range Sciences, Montana State University, Bozeman, MT 59717; 3 Department of Animal Science, “Luiz de Queiroz” College of Agriculture, University of São Paulo, Piracicaba 13418–900, Brazil; 4 Nutricorp, Araras 13601-000, Brazil

**Keywords:** *Bos indicus*, digestibility, feed additives, forage, performance, ruminal parameters

## Abstract

The aim of the present study was to evaluate the inclusion of narasin, salinomycin, or flavomycin for 140 d on ruminal fermentation parameters, apparent nutrient digestibility, and performance of Nellore cattle offered a forage-based diet. In experiment 1, 32 rumen-cannulated *Bos indicus* Nellore steers [initial body weight (**BW**) = 220 ± 12.6 kg] were assigned to individual pens in a randomized complete block design according to their initial shrunk BW. Within block, animals were randomly assigned to 1 of 4 treatments: (1) forage-based diet without feed additives (**CON**; *n* = 8), (2) CON diet plus 13 ppm of narasin (**NAR**; *n* = 8), (3) CON diet plus 20 ppm of salinomycin (**SAL**; *n* = 8), or (4) CON diet plus 3 ppm of flavomycin (**FLA**; *n* = 8). The experimental period lasted 140 d and was divided into 5 periods of 28 d each. The inclusion of feed additives did not impact (*P* ≥ 0.17) dry matter intake (**DMI**), nutrient intake, and apparent total tract digestibility of nutrients. Nonetheless, steers fed NAR had lower (*P* < 0.01) molar proportion of acetate compared with CON, SAL, and FLA steers, whereas ruminal acetate tended to be greater (*P* < 0.09) for SAL vs. CON and FLA, but did not differ (*P* = 0.68) between CON vs. FLA steers. Ruminal propionate was the highest (*P* < 0.01) for steers fed NAR and did not differ (*P* > 0.20) between CON, SAL, and FLA. Consequently, NAR steers had the lowest (*P* < 0.01) Ac:Pr ratio, whereas Ac:Pr did not differ (*P* > 0.18) among CON, SAL, and FLA. Total volatile fatty acids were greater (*P* < 0.04) for NAR and CON vs. SAL and FLA, but did not differ (*P* > 0.67) among NAR vs. CON and SAL vs. FLA. In experiment 2, 164 Nellore bulls (initial shrunk BW = 299 ± 2.5 kg) were assigned to feedlot pens for 140 d in a randomized complete block design. Within block (*n* = 10), animals were randomly assigned to the same treatments used in experiment 1. Average daily gain was greater (*P* < 0.01) in NAR vs. CON, SAL, and FLA bulls, and did not differ (*P* > 0.12) between CON, SAL, and FLA bulls. Bulls fed NAR had greater (*P* < 0.02) DMI (as kg/d or % BW) and final shrunk BW compared with CON, SAL, and FLA bulls, whereas DMI and final shrunk BW did not differ (*P* > 0.26) between CON, SAL, and FLA bulls. Feed efficiency, however, was not impacted (*P* = 0.51) by any feed additives used herein. Collectively, narasin was the only feed additive that benefited performance and ruminal fermentation of Nellore animals fed a forage-based diet.

## Introduction

Worldwide, beef cattle production systems rely largely on forage-based diets as the source of nutrients for meat production. However, seasonal variations in pasture availability, nutritive value, and sward structure of high-forage diets frequently affect nutrient utilization and animal performance by inadequate energy intake (Hills et al., 2015; de Souza et al., 2017) and forage physical effect limiting rumen fill (Conrad et al., 1964; Clark and Armentano, 1997). Feed additives are used as an important nutritional tool to enhance productivity and profitability of beef cattle systems by altering rumen microbiome (Weimer et al., 2008; Schären et al., 2017) and fermentation routes, as well as digestibility and nutrient utilization of the diet (Tedeschi et al., 2003). Nonetheless, the majority of research conducted to date utilizing feed additives focused on high-concentrate diets (Duffield et al., 2012). Yet, little is known about the effects of alternative feed additives on *Bos indicus* cattle fed high-forage diets. Additionally, it is important to establish if the use of feed additive in forage-based diets for 140 d (Rogers et al., 1997; Odongo et al., 2007) would impact the persistence of efficacy, once a diminishing response due to rumen microbial adaptation might occur when feed additives are fed over a prolonged period (Klein et al., 2005).

Narasin is an ionophore that alters rumen fermentation dynamics (Miszura et al., 2018), plasma metabolites by increasing glucose (Sardinha et al., 2020) and reducing urea concentration (Polizel et al., 2020), and improves animal performance (Silva et al., 2015; Polizel et al., 2020). Salinomycin is also an ionophore that improves animal production by favorably altering molar acetate:propionate (**Ac:Pr**) ratio (McClure et al. 1980; Bagley et al., 1988). Flavomycin (bambermycin) is a non-ionophore antibiotic that prevents the synthesis of peptidoglycan on the bacterial cell wall (Volke et al., 1997). Flavomycin might also have indirect benefits on gut tissue protein turnover by suppressing gram-negative pathogenic bacteria (Edwards et al., 2005), as well as gram-positive bacteria which might allow increased dietary fermentation, resulting in a greater propionate molar proportion (Edrington et al., 2003). Although feed additives have similar ruminal modes of action, animal performance might vary depending on dosage, animal, and diet (Bretschneider et al., 2008). Based on this rationale, we hypothesized that supplementation with narasin, salinomycin, or flavomycin would impact nutrient digestibility, change rumen fermentation parameters, and improve productivity of *B. indicus* Nellore cattle fed a forage-based diet. To test this hypothesis, the objective of this experiment was to evaluate the impacts of supplementing narasin, salinomycin, or flavomycin on rumen fermentation characteristics and apparent nutrient digestibility (experiment 1), as well as feed intake, and growth (experiment 2) of *B. indicus* cattle fed a high-forage diet for 140 d.

## Materials and Methods

These studies were conducted at the University of São Paulo, Piracicaba campus (USP/ESALQ; Piracicaba, SP, Brazil; 22º43′31ʺ S, 47º38′51ʺW, and 524 m elevation). Experimental procedures involving animals were reviewed and approved by the Ethics Committee on Use of Animals of School of Veterinary Medicine and Animal Science (University of São Paulo; CEUA/FMVZ; protocol #8582080119).

### Experiment 1: animal metabolism

#### Animals, housing, and diets

Thirty-two rumen-cannulated *B. indicus* Nellore steers [initial body weight (**BW**) = 220 ± 12.6 kg; age = 20 ± 1.0 mo] were assigned to individual pens (concrete-surface; 2 × 2 m, with a feed bunk and waterer) in a randomized complete block design according to their initial shrunk BW. Within block (*n* = 8), animals were randomly assigned to 1 of 4 treatments: (1) forage-based diet without feed additives (**CON**; *n* = 8), (2) CON diet plus 13 ppm of narasin (Zimprova; Elanco Animal Health, São Paulo, Brazil; **NAR**; *n* = 8), (3) CON diet plus 20 ppm of salinomycin (Posistac; Phibro Animal Health Corporation, Guarulhos, São Paulo, Brazil; **SAL**; *n* = 8), or (4) CON diet plus 3 ppm of flavomycin (Flavomycin 80, Huvepharma, Porto Alegre, Rio Grande do Sul, Brazil; **FLA**; *n* =8). The administration rates of NAR, SAL, and FLA used herein were according to manufacturer’s recommendation. The experimental period lasted 140 d and was divided into 5 periods of 28 d each (0, 28, 56, 84, 112, and 140 d).

Throughout the experimental period (days 0 to 140), steers were offered Tifton-85 haylage (*Cynodon dactylon* spp.), which was chopped daily with a vertical mixer (Mixer VM8B, DeLaval International AB, Tumba, Sweden). Haylage average particle length distribution was 50.3 ± 2.5% > 19 mm; 25.8 ± 3.2% >8 mm; 16.1 ± 1.8 % >4 mm; and 7.8 ± 2.0% on bottom sieve according to Penn State Particle Separator procedures (Heinrichs, 1996; Kononoff et al., 2003). Feed additives (NAR, SAL, and FLA) were separately mixed with a 50:50 mixture of ground citrus pulp:ground corn (**CI:C**; 25 g of each ingredient used as a delivery vehicle; as-fed basis). The initial inclusion of feed additive treatment in the 50:50 CI:C mixture was based on a 5.0 kg of forage DMI. Hence, for steers consuming 5.0 kg of forage, the CI:C mixture would contain 65, 100 and 15 ppm of narasin, salinomycin and flavomycin for NAR, SAL and FLA, respectively. Steers from CON group also received the CI:C supplement without the inclusion of feed additives.

Treatments (NAR, SAL, FLA, and CON) were offered to each pen individually and daily prior to haylage feeding to avoid that small amount of supplement would be mixed with hay and compromise intake of the feed additives treatments. Treatment amount were calculated daily based on the previous day individual total forage dry matter intake (**DMI**). From days 0 to 140, animals were fed treatments once daily (0800 hours) and had ad libitum access to haylage (0830 hours), mineral-mixed (offered in separately feed bunk from the haylage and treatments), and fresh water. Steers promptly consumed treatments within 30 min after feeding. The mineral mix (Premiphós 80; Premix; Ribeirão Preto, SP, Brazil) used herein contained 150 g/kg Ca, 80 g/kg P, 12 g/kg S, 134 g/kg Na, 4,500 mg/kg Zn 1,600 mg/kg, 1,400 mg/kg Mn, 800 mg/kg F, 210 mg/kg Co, 180 mg/kg I, and 27 mg/kg Se. The nutritional profile of the haylage and supplement used herein is described in Table 1.

**Table 1.** Nutritional profile of the Tifton-85 (*Cynodon dactylon* spp.) haylage and ground citrus pulp and ground corn (**GC**) mixed used in experiment 1^1^

	Period of study^2^					
Item	1	2	3	4	5	CI:C^3^
Nutrient profile, dry matter basis						
Dry matter	47.7	57.8	64.3	58.1	60.4	87.3
Crude protein, %	18.9	18.0	18.8	16.1	16.0	7.80
NDF, %	63.6	63.0	66.2	69.6	67.2	14.5
ADF, %	30.1	33.3	29.7	34.8	30.8	3.80
Ether extract, %	2.91	2.16	2.48	2.19	2.73	2.60
Ash, %	12.3	11.8	9.29	10.2	10.0	4.91
TDNs^4^, %	53.9	53.8	55.7	54.1	55.5	81.9
Metabolizable energy^5^, Mcal/kg	1.99	1.99	2.06	2.00	2.05	3.10
Net energy of maintence^5^, Mcal/kg	1.10	1.10	1.17	1.11	1.16	2.00
Net energy of gain^5^, Mcal/kg	0.55	0.54	0.61	0.55	0.60	1.35

^1^Based on nutritional profile of each ingredient, which were analyzed via wet chemistry procedures (AOAC, 1990).

^2^The experimental period lasted 140 d and was divided into 5 periods of 28 d each.

^3^CI:C: 50% ground citrus pulp dry and 50% GC.

^4^Calculations were performed according to the equations proposed by Weiss et al. (1992).

^5^Calculated composition using tabular values from NASEM (2016).

#### Sampling, laboratory analyses, and measurements

Samples of haylage and CI:C supplement were collected weekly, pooled across all weeks within each period, and analyzed for nutrient profile (Table 1). From days 23 to 27 (period 1), 51 to 55 (period 2), 79 to 83 (period 3), 107 to 111 (period 4), and 135 to 139 (period 5), total fecal production was individually collected to determine apparent nutrient digestibility. Total fecal production was collected and quantified twice a day using an electronic scale (Marte AC-10K; Marte Cientifica, Sao Paulo, SP, Brazil) at 0800 h and 1800 h, and a representative sample (~10% of wet weight) of the daily production of each steer was collected and stored at −18 °C on the same day of collection. Total tract apparent nutrient digestibility was calculated according to the formula: TTAD (%) = ((DMI × NCDM) – (FDM × NCFM) × 100)/(DMI × NCDM), where TTAD = total tract apparent digestibility, DMI = dry matter intake, NCDM = nutrient content of the DMI (%), FDM = fecal dry matter, and NCFM = nutrient content of the fecal DM (%).

Samples of feed, orts, and feces were dried in a forced-air oven at 60 °C (AOAC, 1990; method #930.15) for 96 hr. Sequentially, the samples were ground through a 1-mm Wiley Mill screen (Marconi, Piracicaba, SP, Brazil). The final DM content was determined after oven-drying the samples at 105 °C for 24 hr (AOAC, 1990; #934.01) and ash concentration was obtained by incinerating the samples in an oven at 550 °C for 4 hr (AOAC, 1990; method #942.05). Sequential detergent fiber analyses were used to determine neutral detergent fiber (**NDF;**Van Soest et al., 1991) and acid detergent fiber (**ADF;**Goering and Van Soest, 1970) with an Ankom 2000 fiber analyzer (Ankom Tech. Corp., Macedon, NY). Sodium sulfite and heat-stable α-amylase were added in the NDF analysis. The extract ether content was determined using an Ankom^XT15^ Extrator (Ankom Technology, Macedon), according to AOAC (1990; method 920.29), using petroleum ether. The total N was determined according to AOAC (1990; method #968.0) using the Leco TruMac N (Leco Corp., St. Joseph, MI) and the crude protein (**CP**) was obtained by multiplying the total N content by 6.25. Calculation of haylage and supplement total digestible nutrients (**TDN**), net energy for maintenance (**NE**_**m**_), and gain (**NE**_**g**_) was performed according to Weiss et al. (1992) and the tabular values proposed by NASEM (2016).

Individual shrunk BW was collected on day 0 after 14 hr of feed and water withdrawal to determine initial BW and to perform the randomization into blocks and treatments. Forage, supplement, and total DMI were recorded daily from each pen by collecting and weighing nonconsumed feed (forage only). Samples of the offered and nonconsumed feed were collected daily from each pen and dried for 24 hr at 105 ± 2 °C in forced-air ovens for dry matter calculation.

On day 0 (immediately prior to the beginning of the experimental period and first treatment offer), 28, 56, 84, 112, and 140 of the experimental period at 0, 6 and 12 hr after CI:C supplement feeding, ruminal fluid samples were manually collected (~100 mL/sample time) by squeezing the ruminal contents into 4 layers of cheesecloth and the ruminal fluid pH was immediately determined (Digimed-M20; Digimed Instrumentação Analítica; São Paulo, SP, Brazil). Approximately 50 mL of the ruminal fluid were collected, pooled across all sampling times (0, 6, and 12 hr), within each experimental period, and stored at −18 °C for subsequent analysis of rumen ammonia and molar proportions of individual volatile fatty acids (**VFAs**; acetate, propionate, butyrate, isobutyrate, valerate, and isovalerate), as well as the Ac:Pr and acetate butyrate:propionate (**AcBut:Pr**) ratios, and total VFA. Frozen ruminal samples were prepared for analysis by thawing, centrifuging (15,000 × *g*) for 60 min at 4 °C, and analyzed for VFA and rumen ammonia according to procedures described by Ferreira et al. (2016) and Broderick and Kang (1980), respectively.

### Experiment 2: animal performance

#### Animals, housing, and experimental design

One hundred and sixty-four *B. indicus* Nellore bulls (initial shrunk BW = 299 ± 2.5; age = 23 ± 3.0 mo) were assigned to pens in a randomized complete block design according to their shrunk BW (after 14 hr of feed and water restriction). The experimental period lasted 140 d, divided into 5 periods of 28 d each. Bulls were kept in a covered feedlot (10 pens per treatment; 4 to 5 bulls per pen; 3 × 6 m) with a concrete floor, feed bunk, mineral bunk, and waterer. Within blocks (*n* = 10), animals were randomly assigned to the same treatments as in experiment 1.

Throughout the experimental period (days 0 to 140), bulls were offered Tifton-85 haylage (*Cynodon dactylon* spp.) which was chopped daily utilizing a vertical mixer (Mixer VM8B, DeLaval International AB). Haylage average particle length distribution was 46.7 ± 3.1 % > 19 mm, 28.1 ± 2.1% > 8 mm, 15.2 ± 2.0 % > 4 mm, and 10 ± 3.8% on bottom sieve according to Penn State Particle Separator procedures (Heinrichs, 1996; Kononoff et al., 2003), whereas ground corn was used as a delivery vehicle for feed additives treatments (NAR, SAL, and FLA). Additionally, animals from CON group also received ground corn with no inclusion of feed additives. Feed additives were mixed into ground corn (200 g/pen for each 5 kg of haylage DMI; as-fed basis) and offered to each pen individually. Bulls promptly consumed the supplement within 30 min after feeding and then the haylage was offered. Treatments were offered daily prior to haylage feeding to avoid that the small amount of concentrate would be mixed with the hay and compromise the intake of feed additives. The nutritional profile of the forage used in the present experiment is described in Table 2.

**Table 2.** Nutritional profile of the Tifton-85 (*Cynodon dactylon* spp.) haylage and **GC** used in experiment 2^1^

	Period of study^2^					
Item	1	2	3	4	5	GC
Nutrient profile, dry matter basis						
Dry matter	37.4	48.0	46.9	49.0	49.7	88.0
CP, %	21.0	23.2	19.3	17.0	13.3	9.18
NDF, %	57.8	56.3	61.3	59.3	61.8	12.6
ADF, %	29.2	30.0	28.7	29.6	34.8	4.59
Ether extract, %	2.54	2.26	3.60	2.40	2.01	3.91
Ash, %	14.1	10.3	13.1	11.1	10.4	1.50
TDNs^3^, %	52.8	54.4	54.4	55.8	55.9	88.9
Metabolizable energy^4^, Mcal/kg	1.96	2.08	2.01	2.06	2.07	3.29
Net energy of maintence^4^, Mcal/kg	1.07	1.18	1.12	1.17	1.17	2.21
Net energy of gain^4^, Mcal/kg	0.51	0.62	0.56	0.61	0.61	1.52

^1^Based on nutritional profile of each ingredient, which was analyzed via wet chemistry procedures (AOAC, 1990).

^2^The experimental period lasted 140 d and was divided into 5 periods of 28 d each.

^3^Calculations were performed according to the equations proposed by Weiss et al. (1992).

^4^Calculated composition using tabular values from NASEM (2016).

From days 0 to 140, animals were fed the treatments (ground corn with or without feed additives) once daily at 0730 hours and had ad libitum access to haylage (0800 hours), mineral–vitamin mix, and fresh water. Mineral mix (Premiphós 80; Premix) used herein was the same as in experiment 1 and was offered separately in feed bunk from haylage and treatments. The initial inclusion of additives in the ground corn was based on a 5.0 kg of forage DMI. Hence, for animals consuming 5.0 kg of forage, the ground corn would contain 65, 100, and 15 ppm of narasin, salinomycin, and flavomycin, for NAR, SAL, and FLA, respectively. The doses of NAR, SAL, and FLA used herein were according to manufacturer’s recommendation. Throughout the experimental period (days 0 to 140), additives dosage offered to the animals was based on the previous day total DMI.

#### Sampling and measurements

At the beginning (day 0) of the experimental period, individual shrunk BW was recorded after 14 hr of feed and water withdrawal to determine animal initial BW and to perform the randomization of the animals into blocks and treatments. To calculate average daily gain (**ADG**) and feed efficiency (**G:F**), bulls were individually weighed on days 0, 28, 56, 84, 112, and 140 (final days of each period) after 14 hr of feed and water restriction. DMI was evaluated daily from each pen within each period by collecting and weighing nonconsumed feed weekly. Hay and total DMI of each pen were divided by the number of bulls within each pen and expressed as kilogram per bull/day. Within each pen, total BW gain and total DMI of each period were used for bull G:F calculation. Samples of feed and orts were collected weekly, pooled across all weeks within each period, and analyzed for nutrient profile as aforementioned for experiment 1.

### Statistical analyses

For all the variables analyzed, animal (experiment 1) or pen (experiment 2) was considered the experimental unit and quantitative data were analyzed using the MIXED procedure of SAS (SAS Inst. Inc., Cary, NC). All data were analyzed using Satterthwaite approximation to determine the denominator df for the test of fixed effects (experiments 1 and 2), with animal(treatment) as random variable for experiment 1. In experiment 2, however, pen(treatment) and animal(pen × treatment) were used as random variables for all variables, except for DMI and G:F that used pen(treatment) as random variables. Model statement for all analyses contained the effects of treatment, day or period, and treatment × day or period interactions and block as independent covariate. The specified term for all repeated statements was day or period, with animal(treatment) as subject for experiment 1, whereas in experiment 2, pen(treatment) was used as subject for DMI and G:F only, and animal(pen × treatment) as subject for all other analyses following the rationale described by St-Pierre (2007) and Bello et al. (2016). The covariance structure used was the first-order autoregressive, which provided the smallest Akaike information criterion and hence the best fit for all variables analyzed. All results from experiment 1 are reported as covariately adjusted least square means for values obtained on day 0, except for forage DMI, and separated using PDIFF. All results from experiment 2 are reported as least square means and were separated using PDIFF. Significance was set at *P* ≤ 0.05 and tendencies were determined if *P* > 0.05 and ≤ 0.10. Results are reported according to the main effects if no interactions were significant.

## Results

### Experiment 1: animal metabolism

Based on manufacturer’s recommendation and previous day forage intake, feed additives consumption during experiment 1 were 13.6 ± 0.2, 20.9 ± 0.3, and 3.1 ± 0.1 mg/kg of DM per day for NAR, SAL, and FLA respectively. Values obtained on day 0 of the study were not significant covariates (*P* > 0.56) for rumen concentrations of acetate, propionate, isobutyrate, butyrate, isovalerate, and valerate, and did not differ among treatments (*P* > 0.28; data not shown), demonstrating that animals were under similar management prior to the beginning of the present study.

No treatment × period interactions were identified for intake and apparent nutrient digestibility (*P ≥* 0.33; Table 3) for steers receiving the experimental treatments. The inclusion of feed additives did not impact (main treatment effect; *P* ≥ 0.17) DMI and specific nutrient intake (Table 3). In addition, there was no effect (*P* ≥ 0.40) on apparent nutrient digestibility among treatments (Table 3). However, there was a period effect (*P* < 0.001) on intake and nutrient digestibility (*P* < 0.01; Table 3), which may be attributed to the variation observed on the quality and composition of forage during the experiment period (Table 1).

**Table 3.** Intake and apparent total tract digestibility of nutrients of *Bos indicus* Nellore steers receiving a high forage-based diets supplemented or not (**CON**, *n* = 8) with narasin (**NAR**, *n* = 8), salinomycin (**SAL**; *n* = 8), or flavomycin (**FLA**; *n* =8) for 140 d

	Treatments^1^					*P*-value^2^		
Item	CON	NAR	SAL	FLA	SEM	Treatment	Period	*T* × *P*
Intake, kg/day								
Dry matter	5.93	5.85	5.71	5.45	0.26	0.20	<0.01	0.33
Organic matter	5.32	5.24	5.09	4.87	0.23	0.17	<0.01	0.39
CP	1.05	1.03	1.01	0.97	0.44	0.20	<0.01	0.43
NDF	3.99	3.94	3.85	3.67	0.17	0.18	<0.01	0.35
ADF	1.88	1.86	1.81	1.73	0.08	0.19	<0.01	0.33
Digestibility, % (dry matter basis)^3^								
Dry matter	52.39	53.14	52.07	53.26	1.08	0.80	<0.01	0.70
Organic matter	57.19	57.86	56.73	58.10	0.96	0.70	<0.01	0.83
Crude protein	63.70	64.31	63.39	63.96	0.93	0.90	<0.01	0.70
NDF	60.27	61.07	58.78	61.06	1.06	0.40	<0.01	0.48
ADF	54.59	55.83	52.90	55.35	1.28	0.40	<0.01	0.58

^1^CON, no feed additives; NAR, inclusion of 13 ppm of narasin (Zimprova, Elanco Animal Health, São Paulo, Brazil); SAL, inclusion of 20 ppm of salinomycin (Posistac, Phibro Animal Health Corporation, Guarulhos, São Paulo, Brazil); FLA, inclusion of 3 ppm of flavomycin (Flavomycin 80, Huvepharma, Porto Alegre, Rio Grande do Sul, Brazil). Within rows, values with different superscripts differ (*P* ≤ 0.05).

^2^ *P*-value for treatment, period, and treatment × period interaction (*T* × *P*).

^3^From days 23 to 27 (period 1), 51 to 55 (period 2), 79 to 83 (period 3), 107 to 111 (period 4), and 135 to 139 (period 5), total fecal production was individually collected to determine apparent nutrient digestibility analysis. Apparent digestibility was calculated according to the formula: TTAD (%) = ((DMI × NCDM) – (FDM × NCFM) × 100) / (DMI × NCDM), where TTAD = total tract apparent digestibility, DMI = dry matter intake, NCDM = nutrient content of the DMI (%), FDM = fecal dry matter, and NCFM = nutrient content of the fecal DM (%).

A treatment × day interaction was only detected (*P* < 0.01) for AcBut:Pr ratio. After the second experimental period, animals fed NAR had the smallest values for AcBut:Pr ratio, whereas it did not differ (*P* > 0.28) between SAL, FLA, and CON. A treatment effect was detected (*P* ≤ 0.02) for molar proportion of acetate, propionate, butyrate, isovalerate, as well as Ac:Pr, and AcBut:Pr ratios. In general, steers fed NAR had lower (*P* < 0.01) molar proportion of ruminal acetate compared with CON, SAL, and FLA steers, whereas ruminal acetate was greater (*P* = 0.04) for SAL vs. CON steers, tended to be greater (*P* = 0.09) for SAL vs. FLA, and did not differ (*P* = 0.68) between CON and FLA steers (Table 4). On the other hand, the molar proportion of propionate was highest (*P* < 0.01; Table 4) for animals fed NAR and did not differ (*P* > 0.20) between CON, SAL, and FLA. Consequently, NAR animals had the lowest (*P* < 0.01) Ac:Pr ratio, whereas Ac:Pr ratio did not differ (*P* > 0.18) between CON, SAL, and FLA (Table 4).

**Table 4.** Molar proportion of rumen VFA, ammonia, and pH of *Bos indicus* Nellore steers receiving a high forage-based diets supplemented or not (**CON**, *n* = 8) with narasin (**NAR**, *n* = 8), salinomycin (**SAL**; *n* = 8), or flavomycin (**FLA**; *n* =8) for 140 d (experiment 1)

	Treatments^1^					*P-*value^2^		
Item	CON	NAR	SAL	FLA	SEM^2^	Treatment	Day	*T* × *D*
Volatile fatty acids, mM/100 mM^3^								
Acetate	73.46^b^	72.98^a^	73.89^c^	73.54^bc^	0.14	<0.01	<0.01	0.34
Propionate	13.77^b^	14.53^a^	13.49^b^	13.43^b^	0.14	<0.01	<0.01	0.17
Isobutyrate	1.01	1.07	1.05	1.07	0.03	0.44	<0.01	0.59
Butyrate	9.05^c^	8.60^a^	8.73^ab^	8.97^bc^	0.10	0.01	<0.01	0.79
Isovalerate	1.52	1.58	1.54	1.66	0.04	0.12	<0.01	0.45
Valerate	1.26	1.23	1.28	1.27	0.02	0.23	<0.01	0.82
Ac:Pr	5.39^b^	5.01^a^	5.49^b^	5.49^b^	0.05	<0.01	<0.01	0.11
AcBut:Pr^4^	5.98^b^	5.65^a^	6.04^b^	6.06^b^	0.05	<0.01	<0.01	<0.01
Total VFA, mM	53.32^a^	51.96^a^	41.11^b^	42.32^b^	3.02	0.02	<0.01	0.81
Ammonia, mg/dL	3.10	2.93	3.38	3.43	0.21	0.29	<0.01	0.53
Rumen pH	6.76	6.89	6.88	6.80	0.05	0.28	<0.01	0.54

^1^CON, no feed additives; NAR, inclusion of 13 ppm of narasin (Zimprova, Elanco Animal Health, São Paulo, Brazil); SAL, inclusion of 20 ppm of salinomycin (Posistac, Phibro Animal Health Corporation, Guarulhos, São Paulo, Brazil); FLA, inclusion of 3 ppm of flavomycin (Flavomycin 80, Huvepharma, Porto Alegre, Rio Grande do Sul, Brazil). Within rows, values with different superscripts differ (*P* ≤ 0.05). Ac:Pr, acetate:propionate ratio; AcBut:Pr, acetatebutirate:propionate ratio

^2^ *P*-value for treatment, day and treatment × day interaction (*T* × *D*).

^3^On day 0 (immediately prior to the beginning of the experimental period and first treatment offer), 28, 56, 84, 112, and 140 of the experimental period at 0, 6, and 12 hr after feeding supplement + treatments, ruminal fluid samples were collected (~100 mL).

^4^Relationship between ketogenic and glucogenic VFA in the rumen as reported by Polizel et al. (2020).

A treatment effect was detected (*P* < 0.01) for molar proportion of butyrate, which was reduced (*P* < 0.01) for animals fed NAR compared with CON and FLA, whereas ruminal butyrate did not differ (*P* > 0.36) between NAR vs. SAL, CON vs. FLA, and tended to be lower (*P* = 0.09) for SAL vs. FLA. A treatment effect was also detected (*P* < 0.01) for total VFA, which was greater (*P* < 0.04) for NAR and CON compared with SAL and FLA, but did not differ (*P* > 0.67) between NAR vs. CON and SAL vs. FLA (Table 4). No treatment effect was detected (*P* > 0.11) for molar proportion of isobutyrate, isovalerate, valerate, ruminal ammonia, and pH (Table 4). A day effect was observed (*P* < 0.01) for all rumen variables herein analyzed (Table 4).

### Experiment 2: animal performance

**Figure 1.**
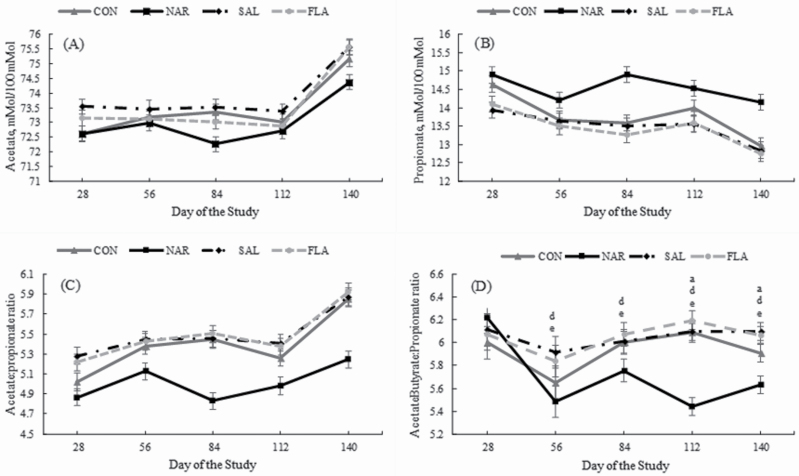
Molar concentration of acetate (Panel A), propionate (Panel B), acetate:propionate ratio (Panel C), and acetatebutyrate:propionate ratio (Panel D) of *Bos indicus* Nellore steers receiving a high forage-based diets supplemented or not (**CON**, *n* = 8) with 13 ppm of narasin (Zimprova; Elanco Animal Health, São Paulo, Brazil; **NAR**, *n* = 8), 20 ppm of salinomycin (Posistac, Phibro Animal Health Corporation, Guarulhos, São Paulo, Brazil; **SAL**, *n* = 8), or 3 ppm of flavomycin (Flavomycin 80, Huvepharma, Porto Alegre, Rio Grande do Sul, Brazil; **FLA**, *n* = 8). Treatments were offered daily throughout the experimental period (days 0 to 140). Rumen samples were collected on day 0 (prior to first treatment administration), 28, 56, 84, 112, and 140 of the study. Data were analyzed using results from day 0 as independent covariate. Within days, letters indicate treatment comparisons (*P* ≤ 0.05): a = CON vs. NAR, b = CON vs. SAL, c = CON vs. FLA, d = NAR vs. SAL, e = NAR vs. FLA, and f = SAL vs. FLA.

Based on manufacturer’s recommendation and previous day forage intake, the feed additives consumption during experiment 2 were 13.1 ± 0.08, 20.1 ± 0.1, and 3.0 ± 0.02 mg/kg of DM per day for NAR, SAL, and FLA respectively (Figure 1).

**Figure 2.**
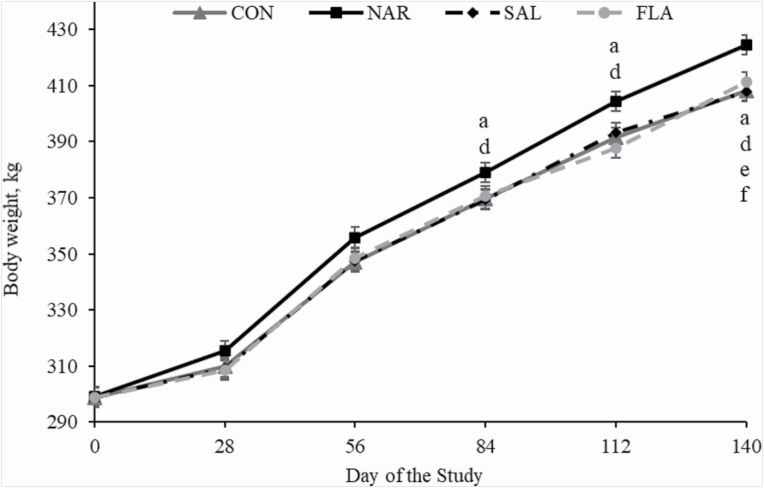
BW of *Bos indicus* Nellore bulls receiving a high forage-based diets supplemented or not (**CON**, *n* = 8) with 13 ppm of narasin (Zimprova; Elanco Animal Health, São Paulo, Brazil; **NAR**, *n* = 8), 20 ppm of salinomycin (Posistac, Phibro Animal Health Corporation, Guarulhos, São Paulo, Brazil; **SAL**, *n* = 8), or 3 ppm of flavomycin (Flavomycin 80, Huvepharma, Porto Alegre, Rio Grande do Sul, Brazil; **FLA**, *n* = 8). Treatments were offered daily throughout the experimental period (days 0 to 140). BW was recorded on day 0 (prior to first treatment administration), 28, 56, 84, 112, and 140 of the study after 14 hr of feed and water withdrawal. Within days, letters indicate treatment comparisons (*P* ≤ 0.05): a, CON vs. NAR, b, CON vs. SAL, c, CON vs. FLA, d, NAR vs. SAL, e, NAR vs. FLA, and f, SAL vs. FLA.

**Figure 3.**
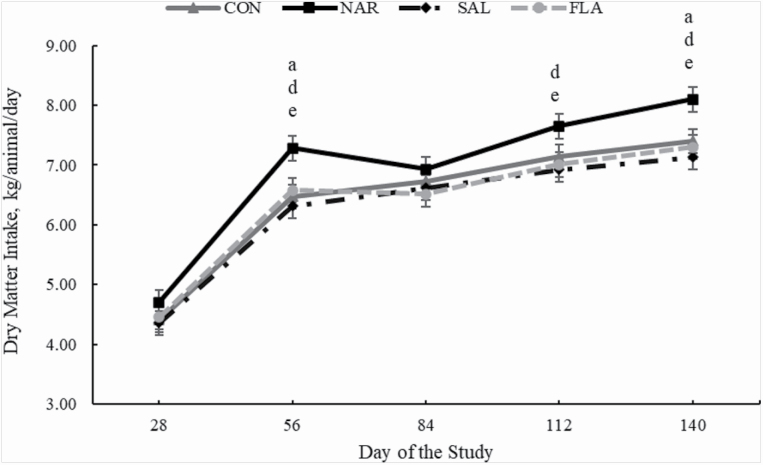
DMI of *Bos indicus* Nellore bulls receiving a high forage-based diets supplemented or not (**CON**, *n* = 8) with 13 ppm of narasin (Zimprova; Elanco Animal Health, São Paulo, Brazil; **NAR**, *n* = 8), 20 ppm of salinomycin (Posistac, Phibro Animal Health Corporation, Guarulhos, São Paulo, Brazil; **SAL**, *n* = 8), or 3 ppm of flavomycin (Flavomycin 80, Huvepharma, Porto Alegre, Rio Grande do Sul, Brazil; **FLA**, *n* = 8). Treatments were offered daily throughout the experimental period (days 0 to 140). DMI was evaluated daily from each pen within each period by collecting and weighing nonconsumed feed weekly. Hay and total DMI of each pen were divided by the number of bulls within each pen and expressed as kilogram per bull per day. Within days, letters indicate treatment comparisons (*P* ≤ 0.05): a, CON vs. NAR, b, CON vs. SAL, c, CON vs. FLA, d, NAR vs. SAL, e, NAR vs. FLA, and f, SAL vs. FLA.

As designed, initial BW did not differ (*P* = 0.94) among treatments (Table 5). During experiment, ADG was greater (*P* < 0.01) in NAR vs. CON, SAL, and FLA bulls, and did not differ (*P* > 0.12) between CON, SAL, and FLA (Table 5; main treatment effect, *P* < 0.01). A treatment × period interaction was detected (*P* = 0.03) for DMI, which was greater (*P* < 0.01) for NAR bulls on periods 2, 4, and 5 of the experiment compared with CON, SAL, and FLA, and did not differ (*P* > 0.26) between CON, SAL, and FLA (Table 5; Figure 3). A tendency was detected (*P* = 0.08; treatment × period interaction) for DMI as % BW, which was also greater (*P* < 0.01) for NAR bulls compared with CON, SAL, and FLA, and did not differ (*P* > 0.26) between CON, SAL, and FLA (Table 5). No treatment effect was detected (*P* = 0.51) for G:F, whereas final shrunk BW was greater (*P* = 0.02, main treatment effect) for NAR animals compared with CON, SAL, and FLA, and did not differ (*P* > 0.52) between CON, SAL, and FLA (Table 5; Figure 2).

**Table 5.** Performance of *Bos indicus* Nellore bulls receiving control (without feed additive; **CON**, *n* = 8), narasin (**NAR**, *n* = 8), salinomycin (**SAL**; *n* = 8), or flavomycin (**FLA**; *n* = 8), in high forage-based diets for 140 d

	Treatments^2^					*P*-value^3^		
Item^1^	CON	NAR	SAL	FLA	SEM	Treatment	Day	*T* × *D*
BW, kg								
Initial (day 0)	298.9	299.2	298.9	298.9	2.50	0.99	**—**	—
Day 28	309.7	314.6	309.1	311.8	2.56	0.41	—	—
Day 56	347.7	354.8	347.2	351.3	2.56	0.13	—	—
Day 84	369.8^b^	378.4^a^	368.8^b^	374.1^ab^	2.56	0.03	—	—
Day 112	391.8^b^	403.9^a^	392.9^b^	397.2^ab^	2.56	<0.01	—	—
Final (day 140)	409.7^b^	424.2^a^	406.4^b^	414.8^b^	2.52	0.02	**—**	**—**
DMI, kg	6.42^b^	6.93^a^	6.26^b^	6.37^b^	0.14	0.04	<0.01	0.03
DMI, % BW	1.82^b^	1.93^a^	1.76^b^	1.78^b^	0.15	0.01	<0.01	0.08
ADG, kg	0.791^b^	0.908^a^	0.812^b^	0.842^b^	0.02	<0.01	<0.01	0.73
G:F, g/kg	119.6	126.6	125.8	127.9	4.14	0.51	<0.01	0.95

^1^On d 0 of the experimental period, individual shrunk BW was recorded after 14 hr of feed and water withdrawal to determine animal initial BW. To calculate ADG and G:F, bulls were individually weighed on days 0, 28, 56, 84, 112, and 140 (final days of each period) after 14 hr of feed and water restriction. Dry matter intake was evaluated daily from each pen within each period by collecting and weighing nonconsumed feed weekly. Hay and total DMI of each pen were divided by the number of bulls within each pen and expressed as kilogram per bull/day. Total BW gain and DMI of each period were used for bull G:F calculation.

^2^CON, no feed additives; NAR, inclusion of 13 ppm of narasin (Zimprova, Elanco Animal Health, São Paulo, Brazil); SAL, inclusion of 20 ppm of salinomycin (Posistac, Phibro Animal Health Corporation, Guarulhos, São Paulo, Brazil); FLA, inclusion of 3 ppm of flavomycin (Flavomycin 80, Huvepharma, Porto Alegre, Rio Grande do Sul, Brazil). Within rows, values with different superscripts differ (*P* ≤ 0.05).

^3^ *P*-value for treatment, day, and treatment × day interaction (*T* × *D*).

## Discussion

Feed additives are used as an important management tool to enhance cattle growth and G:F by altering ruminal fermentative routes, digestibility, and nutrient utilization of the diet (Tedeschi et al., 2003; Duffield et al., 2012). Nonetheless, the majority of research conducted to date with feed additives focused on high-concentrate-based diets (Tedeschi et al., 2003; Duffield et al., 2008, 2012; Ellis et al., 2012) and with monensin or lasalocid as the ionophore, whereas little is known about the effects of others feed additives (ionophore or non-ionophore) on *B. indicus* Nellore cattle fed high-forage-based diets. Moreover, there are limited or inconsistent information about the impacts of feed additives on DMI of forage-based diets (Bretschneider et al., 2008). Given the limited body of research investigating the efficacy and inclusion of ionophores (narasin and salinomycin) or non-ionophore (flavomycin) on ruminal fermentative parameters and performance of Nellore cattle fed high forage-based diets, results from this experiment are also being contrasted with studies using others feed additives and *Bos taurus* cattle.

It is known that the inclusion of ionophores into beef cattle diets alters ruminal fermentation dynamics by changing microbial ecosystem favoring microorganisms, mostly bacteria gram-negative, that are insensitive to the action of ionophores (Tedeschi et al., 2003; Duffield et al., 2012). Most ionophores (lasalocid, monensin, salinomycin, laidlomycin, and narasin) in the market are produced by *Streptomyces* spp. (Nagaraja, 1995) and their mechanisms are similar in the rumen, whereas animal performance might vary depending on dosage, animal, and diet (Nagaraja et al., 1987; Tedeschi et al., 2003; Bretschneider et al., 2008). Narasin, an ionophore used in this study, is produced by the *Streptomyces aureofaciens* and also changes the fermentation dynamics in the rumen toward increased propionate and decreased acetate by affecting gram-positive bacteria on animals fed with high-forage diets (Miszura et al., 2018; Polizel et al., 2020). Salinomycin is also an ionophore antibiotic produced by *Streptomyces albus*, which has been shown to improve animal performance (McClure et al., 1980) by altering acetate:propionate ratio (Bagley et al., 1988), whereas it is still inconclusive in the literature the impacts of this feed additive on forage-fed beef cattle. Flavomycin (bambermycin) is a non-ionophore antibiotic produced by *Streptomyces bambergiensis*, *S. geysirensis*, and *S. ederensis*, which prevents synthesis of peptidoglycan on the bacterial cell wall (Volke et al., 1997) and might have indirect benefits on gut tissue protein turnover by also suppressing gram-negative pathogenic bacteria, such as *Fusobacterium* spp. (Edwards et al., 2005), as well as gram-positive bacteria which might allow increased dietary fermentation, resulting in a greater propionate proportion (Edrington et al., 2003). Moreover, flavomycin may be capable of altering ruminal protozoa population, which in turn might improve fiber digestion (Perry, 2002), and performance of forage-based livestock systems (Beck et al., 2016).

It is still inconclusive in the literature the impacts of ionophores and non-ionophores on nutrient digestibility (Wedegaertner and Johnson, 1983; Ricke et al., 1984; Crossland et al., 2017; Polizel et al., 2020). In the current study, the inclusion of feed additives into forage-based diets did not impact apparent digestibility of nutrients (experiment 1). In agreement with our data, Bell et al. (2017) reported no differences in nutrient digestibility of beef steers receiving forage-based diet with or without monensin. Accordingly, Polizel et al. (2020) also observed no differences on apparent digestibility of nutrients of *B. indicus* Nellore steers receiving forage-based diets with addition or not of narasin. Corroborating our results, Kobayashi et al. (1992) reported no differences in apparent digestibility of nutrients in wethers supplemented with or without salinomycin. Consistent with those findings, Reffett-Stabel et al. (1989) observed that total tract digestibility of nutrients was not affected by salinomycin supplementation when cattle consumed corn-silage based diets. Nevertheless, others have reported that supplementing flavomycin or monensin resulted in similar or reduced dry matter digestibility in beef cattle consuming forage-based diets (DelCurto et al., 1998; Crossland et al., 2017). DelCurto et al. (1998) also reported that total apparent NDF digestibility was not influenced by monensin or flavomycin supplementation when cattle consumed forage-based diets. Accordingly, Flachwosky and Richter (1991) reported that supplementing 0, 5, 10 or 30 mg of flavomycin to beef cattle did not affect nutrient digestibility and rumen fermentation parameters.

Feed additives influence ADG, G:F, and DMI of animals offered a high-concentrate diet (Duffield et al., 2012; Golder and Lean, 2016). Similar results were observed, except for DMI, in animals offered a high-forage diet (Bretschneider et al., 2008). In the current study (experiment 2 only), intake was 7.9%, 8.8%, and 10.7 % greater for animals offered NAR compared with CON, FLA, and SAL, respectively. Similar results were observed when intake was expressed as % of BW. Corroborating our results, Miszura et al. (2019) reported an increase of 7.55% in DMI by adding narasin in high forage-based diets. Conversely, studies reported that inclusion of narasin did not influence forage DMI in animals offered high forage-based diets (Silva et al., 2015; Polizel et al., 2016; Pascoalino et al., 2020). It should be noted that DMI in the present experiment was not depressed by the inclusion of salinomycin in the diet, despite previous research reporting such outcome in forage- (Reffett-Stabel et al. 1989) and feedlot-fed (Owens et al., 1982; Merchen and Berger, 1985; Zinn, 1986a) cattle. DelCurto et al. (1998), however, found that DMI was not influenced by flavomycin, lasalocid, or monensin when steers were fed a forage-based diets. Additionally, Bretschneider et al. (2008) reported that inclusion of ionophores in diets with high inclusion of forage did not affect DMI. The effects of ionophores on DMI might depend on the forage quality consumed by the animals which can influence passage rate and gut fill, and consequently DMI response (Ellis et al. 1984). Nevertheless, the effects of ionophores and non-ionophores additives on DMI of beef cattle consumed high forage-based diets deserve further investigation.

Inclusion of feed additives in beef cattle diets normally influenced G:F by improving or maintaining ADG and reducing DMI (Tedeschi et al., 2003: [Bibr CIT0007][Bibr CIT0014]). In the current study, only narasin improved ADG by 14.8%, 11.8%, and 7.8% compared with CON, SAL, and FLA, respectively, which resulted in heavier animals at the end of the supplementation period. These outcomes are partially resultant from difference in ruminal fermentation parameters in animals supplemented with narasin, given that increasing molar concentration of propionate and total VFA, and decreasing acetate and butyrate in the rumen are positively correlated with greater feed energy utilization and performance (Blaxter, 1962; Russel and Strobel, 1989; McGuffey et al., 2001; Weimer, et al., 2008). Supporting our results, others have also reported increased concentration of rumen propionate and total VFA and reduced concentration of rumen acetate and butyrate when narasin was fed to beef cattle (Miszura et al., 2018; Polizel et al., 2020). Also corroborating this study, flavomycin (Mogentale et al., 2010; Crossland et al., 2017) or salinomycin (Olumeyan et al., 1986; Zinn, 1986b) supplementation did not change ruminal fermentation parameters. In fact, Olumeyan et al. (1986) and Zinn (1986b) reported that rumen fermentation and performance only changed when the diets had increased amount of grain. Accordingly, Rush et al. (1996) reported that steers grazing crested wheatgrass had improved ADG when fed flavomycin, lasalocid, or monensin in a corn-based supplement compared with cattle fed with no additional feed additive. Beck et al. (2016) reported that heifers grazing bermudagrass and tall fescue pastures had improved BW gains when flavomycin or monensin were added in a daily concentrate supplement. Reffett-Stabel et al. (1989) reported that salinomycin and lasalocid supplementation did not affect ADG, but they tended to improve G:F in growing steers fed a corn-silage-based diet. In the present study, only a small amount of grain was used as a delivery vehicle for the additives, and thus results from this experiment should not be associated with the inclusion of grains in the diets. Despite the difference in BW, DMI, and ruminal fermentation parameters herein, they were not sufficient to influence G:F of Nellore bulls consuming a high forage-based diet with the addition of ionophores or non-ionophores additives. One might speculate that the energy density of the diets is the main driver for differences observed between the present study and previous research (Zinn, 1986a, 1986b; Reffett-Stabel et al., 1989; Rush et al., 1996; Beck et al., 2016). Goodrich et al. (1984) summarized that the optimum diet energy density for monensin addition was 2.9 Mcal of ME/kg of diet DM, which is lesser than the values reported in Tables 1 and 2. Hence, the reduced energy intake by animals consuming salinomycin or flavomycin in the present experiment might partially explain the lack of treatment effects on performance.

Similar ruminal pH values were expected, given that all animals consumed forage-based diets and only a small amount of grain was used as a delivery vehicle for the feed additives. Therefore, it is likely that ruminal pH values were maintained in a range that would not impair rumen and cellulolytic bacteria function. Supporting this statement, Bell et al (2017) and Polizel et al. (2020) also reported similar rumen pH values of beef steers offered a high forage-based diet with monensin and narasin, respectively. Accordingly, Crossland et al. (2017) did not observe any effect of flavomycin or monensin supplementation on rumen pH of beef steers offered a forage-based diet. In fact, the ruminal pH in the present study was within a range that supports and maintains adequate fiber digestion in ruminants (Yokoyama and Johnson, 1988).

Feed additives might mitigate ruminal proteolysis and subsequently reduce ammonia synthesis (Goodrich et al., 1984; Rogers et al., 1997). Moreover, rumen ammonia concentrations below 5 mg/dL might limit microbial growth and ruminal fermentation parameters (Satter and Slyter, 1974; Slyter et al., 1979). Feed additives used herein were not capable of affecting ruminal ammonia concentration of beef steers offered a high forage-based diet, despite the permanent impact on ruminal VFA profile. Supporting our data, Bell et al. (2017) demonstrated that supplementation with monensin did not impact rumen ammonia concentration of beef steers fed a high forage-based diet. Similarly, Lemos et al. (2016) reported no difference in rumen ammonia concentration of beef cattle fed a concentrate diet with flavomycin or monensin.

One of the hypotheses of the present study was that feed additives might not have a long-term effect (Guan et al., 2006) and that rumen microbiome adapts to these feed additives (Crossland et al., 2017). Nonetheless, our data demonstrated that only narasin had an impact on rumen fermentation parameters. Accordingly, Polizel et al. (2020) observed an effect of narasin on ruminal parameters of beef cattle fed with high forage-based diets for 140 d. Despite the differences in rumen fermentation, animals fed diet containing narasin had higher and persistent DMI, resulting in heavier animals at the end of the experimental period. Nevertheless, studies are warranted to further understand the benefits of narasin supplementation for an extended period in beef cattle consuming forage-based diets.

Collectively, inclusion of feed additives (ionophore and non-ionophore) in high forage-based diet did not impact nutrient intake and apparent digestibility of nutrients. Conversely, only narasin was able to fully alter rumen VFA profile by impacting the molar concentration of acetate, butyrate, propionate, and total VFA in Nellore steers fed high forage-based diet for a 140-d period. These outcomes might, at least partially, contribute to the improved ADG and final BW of Nellore bulls supplemented with narasin, despite the concurrent increase in DMI. Nonetheless, results from this experiment suggest that supplementing narasin to *B. indicus* Nellore cattle for 140 d might be a feasible alternative to optimize rumen fermentation characteristics and productivity in grazing beef cattle.

## Abbreviations

ADG: average daily gain
BW: body weight
DMI: dry matter intake
G:F: feed efficiency
VFA: volatile fatty acids
